# Apatinib as targeted therapy for sarcoma

**DOI:** 10.18632/oncotarget.24647

**Published:** 2018-05-11

**Authors:** Feng Li, Zhichao Liao, Chao Zhang, Jun Zhao, Ruwei Xing, Sheng Teng, Jin Zhang, Yun Yang, Jilong Yang

**Affiliations:** ^1^ Department of Bone and Soft Tissue Tumor, Tianjin Medical University Cancer Institute & Hospital, Tianjin 300060, People's Republic of China; ^2^ National Clinical Research Center of Cancer, Tianjin Medical University Cancer Institute & Hospital, Tianjin 300060, People's Republic of China; ^3^ Key Laboratory of Cancer Prevention and Therapy, Tianjin 300060, People's Republic of China; ^4^ Tianjin's Clinical Research Center for Cancer, Tianjin 300060, People's Republic of China

**Keywords:** apatinib, sarcoma, targeted therapy, efficacy, safety

## Abstract

Sarcomas are a group of malignant tumors originating from mesenchymal tissue with a variety of cell subtypes. Despite several major treatment breakthroughs, standard treatment using surgery, radiation, and chemotherapy has failed to improve overall survival. Therefore, there is an urgent need to explore new strategies and innovative therapies to further improve the survival rates of patients with sarcomas. Pathological angiogenesis has an important role in the growth and metastasis of tumors. Vascular endothelial growth factor (VEGF) and vascular endothelial growth factor receptors (VEGFRs) play a central role in tumor angiogenesis and represent potential targets for anticancer therapy. As a novel targeted therapy, especially with regard to angiogenesis, apatinib is a new type of small molecule tyrosine kinase inhibitor that selectively targets VEGFR-2 and has shown encouraging anticancer activity in a wide range of malignancies, including gastric cancer, non-small cell lung cancer, breast cancer, hepatocellular carcinoma, and sarcomas. In this review, we summarize the preclinical and clinical data for apatinib, focusing primarily on its use in the treatment of sarcomas.

## INTRODUCTION

Sarcomas are a group of heterogeneous malignant tumors derived from mesenchymal tissue [1]. These malignancies, including more than 50 subtypes, have unique clinical and histologic features [1]. Although sarcomas are less common than other tumors, they account for almost 21% of all solid tumors in children and are the third leading cause of cancer-related death among people under 20 years [1, 2]. In recent years, despite significant developments in new multidisciplinary therapies, such as surgery, chemotherapy and radiation therapy, the 5-year survival rate has remained relatively unchanged [3]. This phenomenon is particularly evident in metastatic or recurrent advanced disease, with a median overall survival (mOS) of about 15 months and with only 10% of patients still alive at 5 years [4]. Therefore, there is an urgent need to develop new strategies and explore innovative therapies to further improve the survival rate of patients with sarcomas.

Angiogenesis is an important stage of tumor growth and metastasis [5–9]. Vascular endothelial growth factor (VEGF) plays an important role in angiogenesis, and VEGF receptors (VEGFRs) are tyrosine kinase (TK) receptors and the key regulators of this process [10, 11]. VEGFRs activate downstream signaling of the phospholipase Cγ-protein kinase CMAP kinase pathway, but not the Ras pathway, leading to cell proliferation [11, 12]. The VEGFR family of proteins includes three high-affinity TK receptors, namely Flt-1/VEGFR-1, Flk-1/KDR/VEGFR-2, and Flt-4/VEGFR-3 [11]. Both VEGFRs contribute to pathological angiogenesis, either directly or through the stimulation of migration/activation of macrophage lineage cells to stimulate tumor growth and metastasis [11]. VEGFR-1 (Flt-1) has very high affinity for VEGF (Kd = 1-10 × 10^−12^ M) but about 10-fold less kinase activity than VEGFR-2 [11]. VEGFR-2 has TK activity approximately one order of magnitude greater than that of VEGFR-1, and the knockout of the *VEGFR-2*/*flk-1* gene in mice has shown that it is the major positive signal transducer in angiogenesis [11, 13, 14]. The VEGF-C/D and VEGFR3 systems mostly regulate lymphangiogenesis [11, 15]. Thus, VEGFRs represent potential targets for tumor-targeted therapy, especially VEGFR-2 [11]. Therefore, the inhibition of VEGFR-2 activity by specific targeted inhibitors of VEGFR-2 is a promising strategy for inhibiting tumor angiogenesis [16]. Recently, several VEGFR-2 inhibitors, including receptor specific antibodies and small molecules such as sorafenib, vandetanib, cediranib, and sunitinib, have been rapidly developed and have achieved good results in clinical testing [17–19]. Sarcoma is one indication for VEGF/VEGFR targeted therapy, based on its effects on angiogenesis and the observation that overexpression of VEGFRs, particularly VEGFR-2, and is significantly associated with low survival rates in patients with sarcomas [16, 20–23].

Apatinib, also known as YN968D1, is a novel, oral, small molecule TK inhibitor of VEGFR-2 [24]. Preclinical and clinical studies have shown that apatinib has promising efficacy and manageable side effects in the treatment of a variety of solid tumors [24–30]. Especially in advanced gastric cancer, apatinib has been shown to significantly prolong the survival time of patients after standard chemotherapy has failed, and can effectively improve treatment compliance [27, 31]. Based on these findings, apatinib was approved by the China Food and Drug Administration (CFDA) in 2014 as a subsequent-line treatment for patients with advanced gastric cancer. To date, several phase II/III clinical trials of apatinib for the treatment of various cancers, such as non-small cell lung cancer (NSCLC), breast cancer, hepatocellular carcinoma, and sarcomas, have been completed or are ongoing [28–30, 32].

### Preclinical findings for apatinib

*In vitro*, apatinib has demonstrated anti-angiogenic properties including the inhibition of budding rat aortic rings, the proliferation and migration of human umbilical vein endothelial cells, and tube formation induced by VEGF. The inhibition of cell migration and vessel formation was similar to that observed for sunitinib. In addition, apatinib suppressed the phosphorylation of VEGFR-2/KDR and ERK1/2, a downstream of VEGF signaling, in a concentration-dependent manner at the cellular level [25, 33].

*In vivo*, once-daily oral administration of apatinib produced a statistically significant, dose-dependent inhibition of tumor growth in established NCI-H460 human lung tumors, HCT-116 human colon tumors, and SGC-7901 human gastric tumors compared with control nude mice [25]. In addition, apatinib appeared to exert synergistic effects when assessed in combination with docetaxel or doxorubicin in lung cancer models, and with oxaliplatin or 5-FU in colon cancer models [25]. Apatinib also appeared to reverse multidrug resistance by inhibiting the transport function of multidrug resistance protein 1 (ABCB1), multidrug resistance-associated protein 1 (MRP1, ABCC1), and breast cancer resistance protein (BCRP, ABCG2) [34, 35]. This suggests that apatinib may act synergistically with standard chemotherapy agents in the treatment of certain malignancies. Tong et al. showed that the addition of apatinib to standard chemotherapy improved tumor inhibition of ABCB1-overexpressing leukemia cell lines [34].

**Figure 1:**
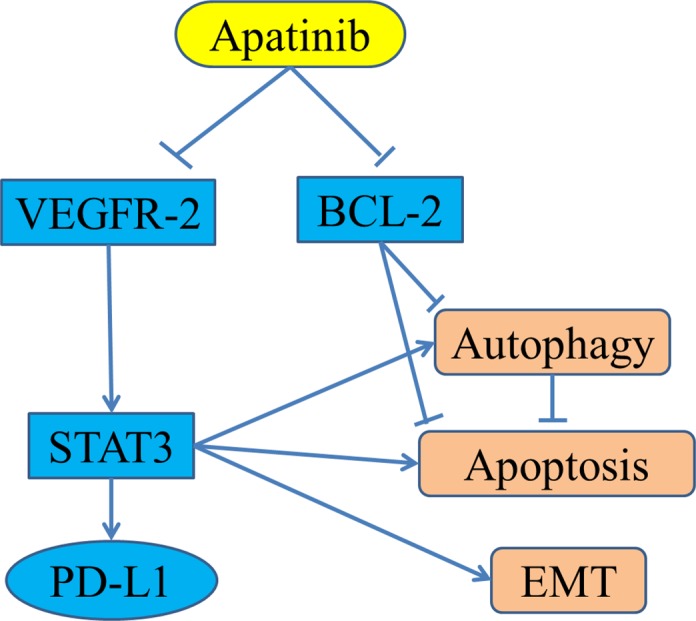
The reported anti-cancer mechanisms of apatinib in osteosarcoma

As for the preclinical data of apatinib in sarcoma field, there are two important studies reported by Prof. Wei Guo group. The first study reported by Liu K et al. shows that apatinib can inhibit the growth of osteosarcoma *in vivo* and *in vitro* [23]. *In vitro*, apatinib can inhibit osteosarcoma growth by inducing autophagy and apoptosis of osteosarcoma cells. The mechanism is that apatinib can directly inhibit the expression of Bcl-2, an anti-apoptotic gene, and inactivate signal transducer and activator of transcription 3 (STAT3) mediated by VEGFR2 (Figure 1). Interestingly, the effect of apatinib on apoptosis of osteosarcoma cells was enhanced by inhibition of autophagy. This suggests that autophagy is a protective factor in the treatment of osteosarcoma with apatinib. *In vivo*, apatinib also reduced the volume of osteosarcoma compared with controls. This study suggests that the combined use of autophagy inhibitors may enhance the antitumor activity of apatinib. The second study reported by Zheng B, et al. that apatinib attenuates migration and invasion by suppressing epithelial-mesenchymal transition (EMT) and inactivating STAT3. Furthermore, apatinib reduces PD-L1 expression in osteosarcoma cells (Figure 1). [36] So these data suggest that apatinib might effect as not only a target therapeutic drug, but also an immunotherapy modulator for sarcoma patients.

### Clinical trials of apatinib for epithelial tumors

The efficacy of apatinib for the treatment of a variety of solid tumors has been investigated in several phase I–III clinical trials in gastric cancer, [27, 31] NSCLC, [28] triple-negative breast cancer, [29] non-triple-negative breast cancer, [32] advanced solid tumors, [24] and advanced hepatocellular carcinoma [30] (Summarized in Table 1).

**Table 1:** Clinical trials of apatinib for molecular targeted therapy in tumors

Tumor type	Trial	Enrollment	Outcomes (apatinib vs. placebo)	AEs
Gastric cancer [27, 31]	Phase II	144 (Placebo: n=48, apatinib 850mg QD: n= 47, apatinib 425mg BID: n=46)	mPFS: 3.67(850mg QD) vs. 1.40 months, 3.20(425mg BID) vs. 1.40 months, mOS: 4.83(850mg QD) vs. 2.50 months, 4.27 (425mg BID) vs. 2.50 months	Toxicities were generally well tolerated.
	Phase III	270(apatinib:180 vs. placebo:90)	mOS: 195 vs. 140 days, mPFS: 78 vs. 53 days, ORR: 2.84% vs. 0.00%	Treatment of apatinib group was generally well tolerated.
Non-small-cell lung cancer [28]	Phase II	135(apatinib:90 vs. placebo: 45)	mPFS: 4.7 vs. 1.9 months, RR: 12.2% vs. 0%, DCR: 68.9% vs. 24.4%.	AEs were generally mild or moderate in severity and were manageable
Non-triple-negative breast cancer [29]	Phase II	38	mPFS: 4.0 months, ORR: 16.7%, DCR: 66.7%, mOS: 10.3 months.	Most toxicity was mild and manageable. Grade 3: 16.6%.grade 4:0%.
Triple-negative breast cancer [32]	Phase II	84(IIa:25, IIb:59)	Phase IIa: mPFS: 4.6 months, OS: 8.3 months; Phase IIb: ORR: 10.7%, CBR: 25.0%, mPFS: 3.3 months, OS: 10.6 months.	Phase IIa (750 mg/day): grade 4: 3(12%); Phase IIb (500 mg/day): grade 4: 2(3.4%)
Advanced solid tumors [24]	Phase I	46	MTD: 850 mg qd Recommended dose: 750 mg qd	Treatment-related AEs were generally mild or moderate in severity and were manageable.
advanced hepatocellular carcinoma [30]	Phase II	121	mTTP(850mg): 4.2 months, mOS(850mg): 9.7 months, DCR(850mg): 48.57%; mTTP(750mg): 3.3 months, mOS(750mg): 9.8 months, DCR(750mg): 37.25%.	Apatinib has been well tolerable in patients; most of the adverse event could be managed by dose interruptions or reductions.

Abbreviations: mPFS, median progression-free survival; mOS, median overall survival; ORR, objective response rate; RR, response rate; DCR, disease control rate; CBR, clinical benefit rate; MTD, maximum tolerated dose; mTTP, median time to progression; BID, twice a day; QD, once a day.

A phase I clinical trial was conducted by Li et al. to determine the maximum tolerated dose (MTD), safety profile, pharmacokinetic variables, and antitumor activity of apatinib in advanced solid malignancies (NCT00633490) [24]. The MTD was determined to be 850 mg once daily and a dose of 750 mg once daily was recommended. With respect to efficacy, partial remission (PR) was observed in 7% of patients and stable disease (SD) in 24%, and the disease control rate (DCR) was 83.8% (7+24/37). The safety profile was acceptable and the regimen was found to be well tolerated.

**Figure 2:**
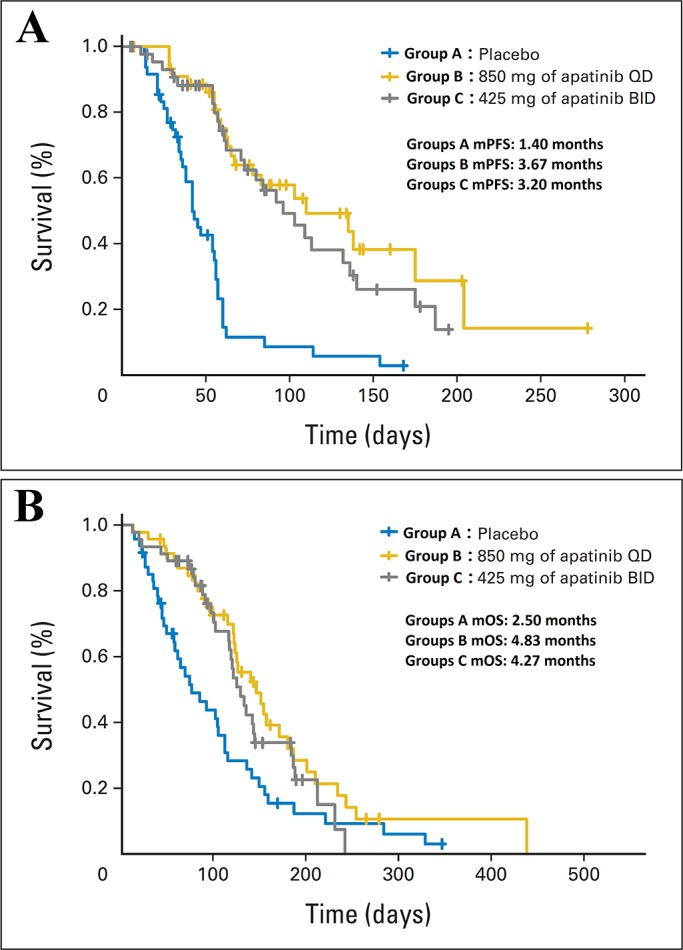
The efficacy evaluation of apatinib in the phase II clinical trial in patients with advanced metastatic gastric cancer **(A)** Kaplan-Meier estimates of progression-free survival (PFS). **(B)** Kaplan-Meier estimates of overall survival (OS). BID, twice a day; QD, once a day. (Cited from: Li J, Qin S, Xu J, Guo W, Xiong J, Bai Y, et al. Apatinib for chemotherapy-refractory advanced metastatic gastric cancer: results from a randomized, placebo-controlled, parallel-arm, phase II trial. J Clin Oncol. 2013; 31: 3219-25. doi: 10.1200/JCO.2013.48.8585.)

**Figure 3:**
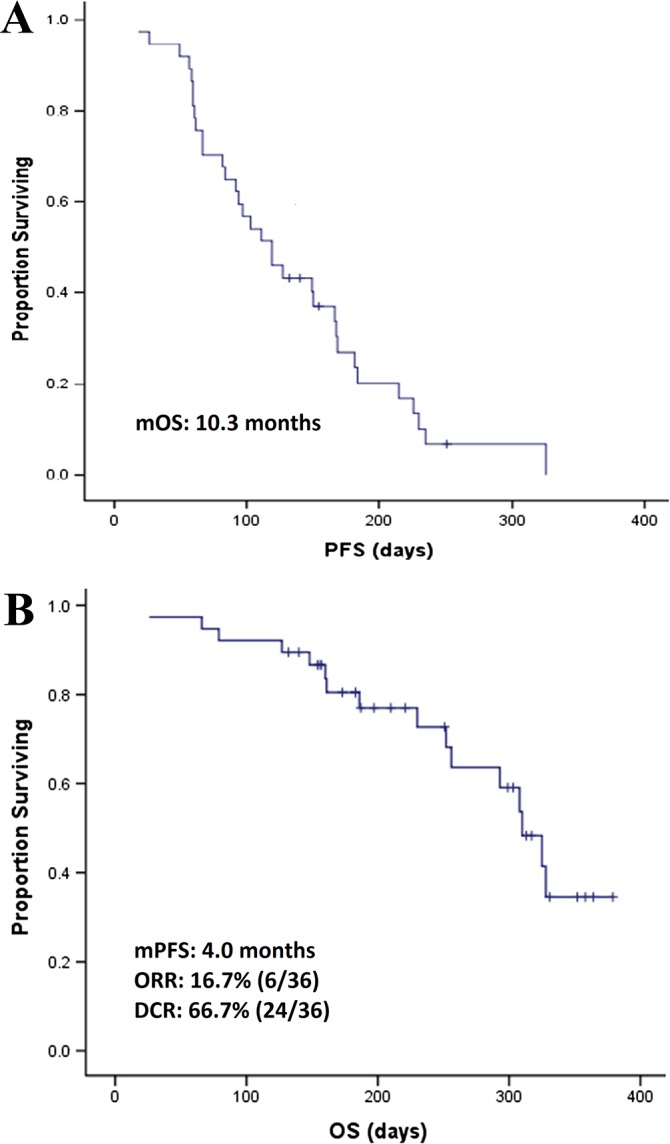
The efficacy evaluation of apatinib in the phase II clinical trial in patients with non-triple-negative metastatic breast cancer **(A)** Kaplan-Meier estimates of progression free survival (PFS). **(B)** Kaplan-Meier estimates of overall survival (OS). (Cited from: Hu X, Cao J, Hu W, Wu C, Pan Y, Cai L, et al. Multicenter phase II study of apatinib in non-triple-negative metastatic breast cancer. BMC Cancer. 2014; 14: 820. doi: 10.1186/1471-2407-14-820.)

**Figure 4:**
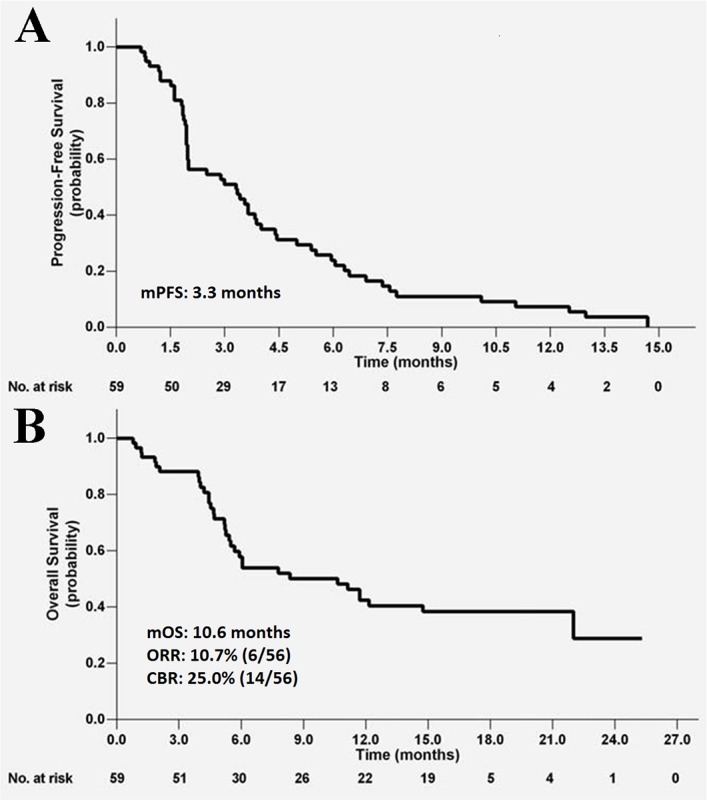
The efficacy evaluation of apatinib in the phase II clinical trial in patients with metastatic triple-negative metastatic breast cancer **(A)** Kaplan-Meier estimates of progression free survival (PFS). **(B)** Kaplan-Meier estimates of overall survival (OS).(Cited from: X, Zhang J, Xu B, Jiang Z, Ragaz J, Tong Z, et al. Multicenter phase II study of apatinib, a novel VEGFR inhibitor in heavily pretreated patients with metastatic triple-negative breast cancer. Int J Cancer. 2014; 135: 1961-9. doi: 10.1002/ijc.28829.)

Phase II clinical trials of apatinib have been performed in patients with gastric cancer, breast cancer, NSCLC, and hepatocellular carcinoma to evaluate the efficacy and safety of apatinib in heavily pretreated patients with malignant tumors. In the trial in gastric cancer, [27] 144 patients were enrolled and divided into three groups: group A, placebo; group B, 850 mg of apatinib once daily; and group C, apatinib 425 mg twice daily. Median progression-free survival (mPFS) for groups A, B, and C was 1.40 months, 3.67 months, and 3.20 months, respectively; and mOS was 2.50 months, 4.83 months, and 4.27 months, respectively. A statistically significant difference in PFS and OS was observed between the apatinib and placebo groups (P <0.001), but no difference was found between the two apatinib groups (Figure 2). In the study of non-triple-negative metastatic breast cancer, mPFS for the 38 enrolled patients was 4.0 months, [32] the objective response rate (ORR) was 16.7% (6/36), DCR was 66.7% (24/36), and mOS were 10.3 months (Figure 3). In the study of triple-negative metastatic breast cancer in 59 patients, [29] mPFS and mOS were 3.3 months and 10.6 months, respectively (Figure 4). In the 56 evaluable patients, ORR and clinical benefit rate (CBR) were 10.7% and 25.0%, respectively. In the study of NSCLC, 135 patients (90 in the apatinib arm, 45 in the placebo arm) were included, [28] and mPFS, response rate (RR), and DCR were significantly better in the study arm (4.7 months, 12.2%, and 68.9%, respectively) than in the placebo arm (1.9 months, 0%, and 24.4%, respectively). In the study of hepatocellular carcinoma, 144 patients were enrolled and assigned to stage 1 (36 patients, 850 mg once daily) and stage 2 (85 patients, 750 mg once daily) [30]. Median time to progression (mTTP), mOS, and DCR in the stage 1 group were 4.2 months, 9.7 months, and 48.57% respectively; in the stage 2 group were 3.3 months, 9.8 months, and 37.25%, respectively.

**Figure 5:**
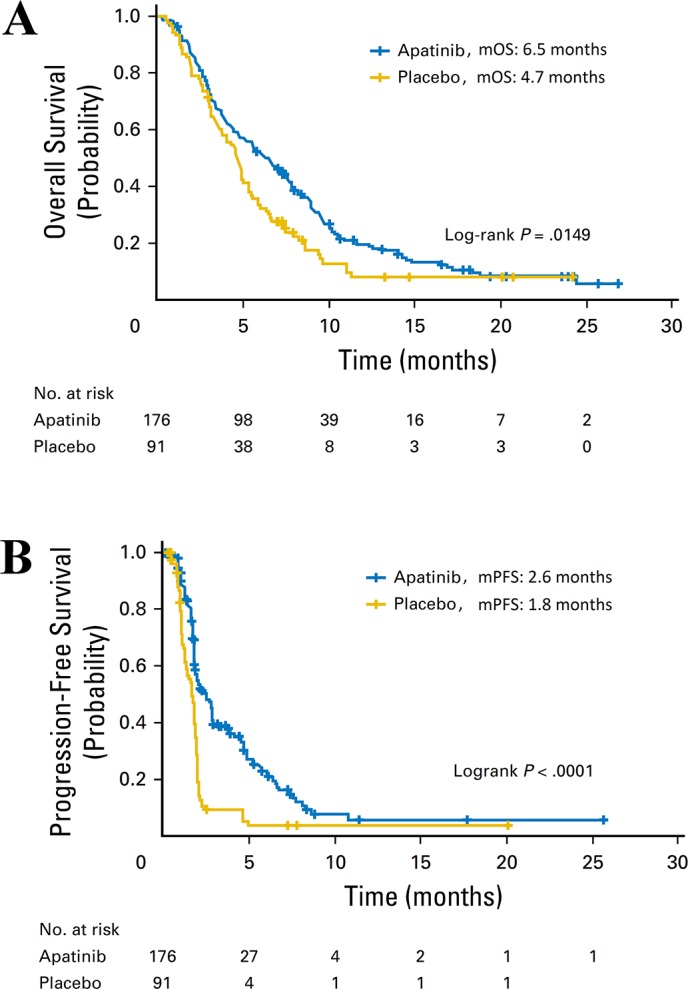
The efficacy evaluation of apatinib in the phase III clinical trial in patients with advanced gastric cancer or gastroesophageal junction adenocarcinoma **(A)** Kaplan-Meier estimates of overall survival (OS). **(B)** Kaplan-Meier estimates of progression free survival (PFS). (Cited from: Li J, Qin S, Xu J, Xiong J, Wu C, Bai Y, et al. Randomized, Double-Blind, Placebo-Controlled Phase III Trial of Apatinib in Patients With Chemotherapy-Refractory Advanced or Metastatic Adenocarcinoma of the Stomach or Gastroesophageal Junction. J Clin Oncol. 2016; 34: 1448-54. doi: 10.1200/JCO.2015.63.5995.)

A phase III trial was conducted by Li et al. to evaluate the efficacy and safety of apatinib in 267 patients with advanced gastric cancer or gastroesophageal junction adenocarcinoma who had received prior chemotherapy [31]. The outcomes demonstrated that mOS and mPFS of the apatinib arm were significantly prolonged compared with placebo (6.5 months vs. 4.7 months, and 2.6 months vs. 1.8 months, respectively) (Figure 5).

These clinical trial results indicate the therapeutic efficacy and manageable adverse effects (AEs) of apatinib in epithelial tumors.

### Effectiveness of apatinib in case reports of sarcomas

To the best of our knowledge, a total of six case reports regarding the treatment of sarcoma with apatinib have been published, and the details of these reports are summarized in Table 2.

**Table 2:** Case reports for apatinib as a molecular targeted therapy for sarcoma

Patient	Age	Sex	Histology	Dose (mg)	Efficacy	AEs
1 [37]	78	Male	MFH	500	PR, PFS: 6 months	grade 2: skin rash, short-lived elevated alanine transaminase and aspartate amino transferals
2 [38]	68	Female	round cell liposarcomas	500	PR, PFS: 6 months	grade 1: elevated transaminase grade 2: hypertension, thrombocytopenia
3 [39]	74	Male	angiosarcomas	500	PR, PFS: 12 months	mild HFS
4 [40]	50	Male	osteosarcomas	500	PR	mild HFS, slight high blood pressure
5 [41]	18	Male	ASPS	500	PR, PFS: 12 months	grade 2: skin rash, short-term elevated alanine transaminase and aspartate amino transferals grades 3-4 : HFS
6 [42]	81	Female	PLS	425	PR, PFS: 3 months	grade 2 hypertension, grade 3 HFS

Abbreviations: MFH, malignant fibrous histiocytoma; ASPS, alveolar soft part sarcomas; PR, partial remission; HFS, hand–foot syndrome; PFS, progression-free survival.

Of the six case reports, five patients were given apatinib daily 500mg, and one patient was 425mg per day. The final efficacy of all these six patients was evaluated as PR. The main AEs were grade 1–4 non-hematologic toxicities, including skin rash, mild hand-foot syndrome (HFS), hypertension, and transient elevation of aminotransferase and aspartate aminotransferase, which were adequately controlled after symptomatic treatment or reduce the dose. No serious drug-related side effects were observed. Ji et al. reported a 78-year-old male patient with malignant fibrous histiocytoma (MFH) who had multiple lung metastases a year after surgery and symptoms of sputum and hemoptysis [37]. After two cycles of targeted treatment, expectoration was markedly reduced, without hemoptysis. The size of the lung metastasis was significantly reduced and was considered PR. At the time of the report, 6-month PFS had been achieved. Dong et al. described a 68-year-old Chinese female patient initially diagnosed with advanced multiple abdominal and pelvic cavity round cell liposarcomas, accompanied with liver metastasis [38]. After nearly 1 month of treatment with apatinib, the middle and right abdominal mass had diminished and the metastatic lesion in the liver remained stable, and the patient was considered to be in PR. At the time of the report, 6-month PFS had been achieved. Ji et al. reported a 74-year-old male patient with angiosarcomas of the scalp, and 2 months after surgery, whole-body positron emission tomography/computerized tomography confirmed local tumor recurrence with multiple metastases in the right temporal lobe, subcutaneous adipose tissue, and bilateral lung lobar [39]. After 1 cycle of treatment with apatinib, the volume of multiple pulmonary metastases had significantly decreased or even disappeared, and the recurrent tumor of the scalp had completely disappeared and the patient was considered to be in PR. The PFS was 12 months. A 50-year-old male patient diagnosed with osteoblastic osteosarcomas was reported by Zhou et al. [40]. Although the patient had undergone multiple resections, he developed multiple pulmonary metastases. After 11 months of targeted therapy with apatinib, a total of nine lesions disappeared and no new lesions were observed, so he was evaluated as in PR. Zhou et al. also described a case of an 18-year-old male patient with alveolar soft part sarcomas (ASPS) and multiple pulmonary metastases at the initial visit [41]. After 1 month of treatment, the size and number of pulmonary metastases decreased, and almost all metastatic lesions had disappeared by 3 months of treatment. The patient was evaluated as in PR, and 12-month PFS had been achieved at the time of the case report. Zhou et al. presented an 81-year-old Chinese woman with advanced pleomorphic liposarcomas (PLS) who received apatinib 425mg per day after failure chemotherapy [42]. One week later, symptoms such as abdominal distension, nausea and vomiting gradually eased. CT examination showed that the tumor was slightly reduced and the internal necrosis area increased. The case was eventually evaluated as PR and received a 3-month PFS.

The results of these studies show that apatinib is efficacious and its adverse effects are manageable in the treatment of malignant sarcoma and thus represent a promising treatment option for patients with metastatic or recurrent sarcomas.

### Safety and efficacy of apatinib in sarcoma treatment: two retrospective studies

Two retrospective studies of apatinib for the treatment of sarcomas have been conducted to date, to the best of our knowledge.

**Figure 6:**
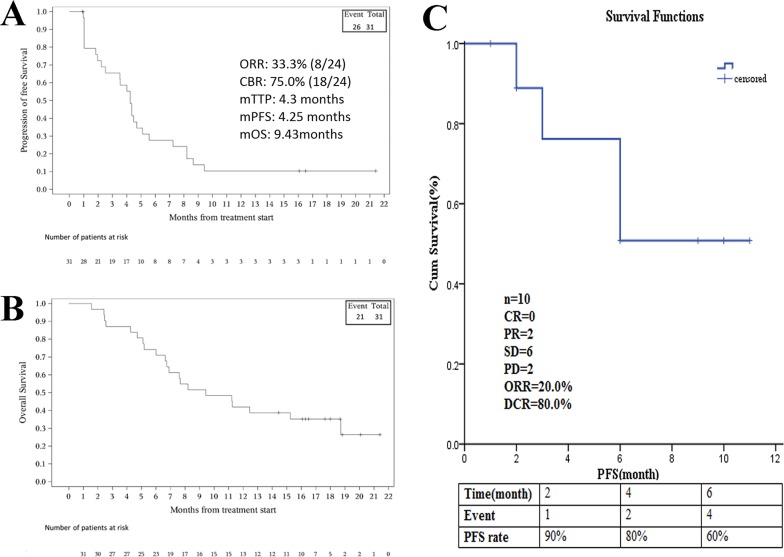
The efficacy evaluation of apatinib in two retrospective studies in patients with advanced sarcoma **(A)** Kaplan-Meier estimates of progression free survival (PFS). **(B)** Kaplan-Meier estimates of overall survival (OS). **(C)** Kaplan-Meier estimates of progression free survival (PFS). (Cited from: Zhu B, Li J, Xie Q, Diao L, Gai L, Yang W. Efficacy and safety of apatinib monotherapy in advanced bone and soft tissue sarcoma: an observational study. Cancer Biol Ther. 2017: 0. doi: 10.1080/15384047.2017.1416275; Li F, Liao Z, Zhao J, Zhao G, Li X, Du X, et al.. Efficacy and safety of Apatinib in stage IV sarcomas: experience of a major sarcoma center in China. Oncotarget. 2017. doi: 10.18632/oncotarget.16293.)

The medical records of 31 patients who received apatinib between September 2015 and August 2016 were retrospectively reviewed by Yang et al. to evaluate short-term efficacy, mTTP, and safety [43]. Nineteen (61.3%) patients received apatinib after failing second-line or further cytotoxic chemotherapy, 8 (25.8%) patients received apatinib as second-line therapy, and 4 (12.9%) patients who refused chemotherapy were given apatinib as first-line therapy. In the study cohort, one patient was treated with apatinib as adjunctive therapy after ablation, while the remaining 30 received apatinib as salvage therapy. AEs led to treatment discontinuation in 3 patients, and 3 more discontinued for personal reasons. Among the 24 remaining patients eligible for evaluation of tumor response to apatinib, 25 patients were available for survival analysis. The ORR was 33.3% and the CBR was as high as 75.0%. The mTTP was 4.3 months (range, 1.8–11.6 months). The median PFS of all patients was 4.25 (95% CI, 2.22–5.11) months, while the median OS was 9.43 (95% CI, 6.64–18.72) months (Figure 6A-6B) [44]. Most AEs were of grade 1 or 2, and grade 3 AEs included hypertension (n = 2, 6.5%), HFS (n = 2, 6.5%), and diarrhea (n = 1, 3.2%). No grade 4 AEs or drug-related mortality occurred.

The other retrospective analysis was conducted by Li et al.,[45] with the aim of evaluating the efficacy and safety of apatinib in patients with stage IV sarcomas who had failed to respond to previous standard chemotherapy. Data were examined from 16 patients treated with apatinib as salvage treatment at a dose of 500 mg/d; 28 days per cycle. Patients underwent an average duration of 3.2 cycles (range 0–9 cycles). Median follow-up time was 8.4 months (1–12 months). Of the 16 patients evaluated, 10 received apatinib treatment more than one cycle, so these 10 patients were evaluated for efficacy. The mPFS was 8.84 months, and in the final efficacy evaluation 2 patients were evaluated as having a PR, 6 patients had SD and the remaining 2 patients had progressive disease (PD). (Figure 6C) The ORR was 20.0% (2/10) and the DCR was 80.0% (8/10). The most common grade 3–4 treatment-related AEs were hypertension (n = 3, 18.7%), HFS (n = 2, 12.5%), and proteinuria (n = 1, 6.3%). No drug-related severe AEs were reported.

In terms of efficacy, the study by Yang et al. had a better ORR than the study by Li et al. (33.3% vs. 20%); however, mPFS and DCR were worse (4.3 months vs. 8.84 months; 75.0% vs. 80.0%, respectively). These studies show subtle differences in efficacy, possibly arising from differences in the cohort sizes. Toxicities were similar between the studies, and were mainly mild to moderate non-hematologic toxicities including grade 1–2 skin rash, mild HFS, hypertension, and transient elevation of aminotransferase and aspartate aminotransferase, which were well controlled after symptomatic treatment. No serious drug-related side effects were observed. In short, as these retrospective studies included small study populations, they support only a preliminary role of apatinib for the treatment of sarcoma. To further confirm the effect of apatinib in this type of cancer, phase II/III clinical trials are required.

### Safety and efficacy of apatinib compared with other anti-angiogenic drugs for the treatment of sarcoma

Despite a lack of phase II/III clinical trials of apatinib for sarcoma, the preliminary studies described above, with mPFS of 4.3 and 8.84 months, support the potential superiority of apatinib over other targeted anti-angiogenesis drugs for sarcoma, including pazopanib, sunitinib, and sorafenib. Sleijfer et al. conducted a phase II trial of pazopanib in 142 patients with relapsed or refractory advanced soft tissue sarcomas (STS). The study population was divided into four groups according to disease category: adipose cell STS, leiomyosarcomas, synovial sarcomas, and other STS types. PFS in the groups was 2.9 months, 3.3 months, 5.8 months, and 3.3 months, respectively [46]. A phase II, multicenter clinical trial of sunitinib in 53 patients with advanced non-gastrointestinal stromal tumor soft tissue sarcomas was reported by George et al. One patient achieved confirmed PR and 10 patients (20%) achieved SD for at least 16 weeks [47]. In a phase II clinical study of sorafenib for metastatic or recurrent sarcomas, 147 patients were enrolled. The median follow-up time was 6 months and PFS was 3.2 months [48]. The most common side effects of these reported targeted anti-angiogenesis agents include hand-foot skin reaction, hypertension, proteinuria, rash, diarrhea, hyperbilirubinemia, rash/desquamation, fatigue, thrombocytopenia, leukopenia, diarrhea, nausea, vomiting and so on [46–48]. The toxicity of apatinib appears to be similar to that of other targeted anti-angiogenesis agents, with no serious adverse events reported.

### Ongoing trials of apatinib for sarcomas

Based on clinical trials for apatinib in epithelial tumors and retrospective analysis and case reports for sarcomas, a number of phase II/III clinical trials of apatinib for sarcoma have been initiated in China. Tianjin Medical University Cancer Institute and Hospital has registered a prospective, open-label, single-arm, multicenter phase II trial to evaluate the efficacy and safety of apatinib for chemotherapy failure IV stage STS, with an estimated enrollment of 80 patients. The clinical trial registered by Shanghai Jiao Tong University Affiliated Sixth People's Hospital is also expected to enroll 80 patients, with the purpose of exploring the safety and effectiveness of apatinib for advanced STS. Peking University People's Hospital has registered two clinical trials, a one-armed, phase II, open-label, multicenter prospective trial of apatinib for advanced STS patients after failure of traditional therapy, and a single arm, phase II/III, single-center trial of apatinib for advanced osteosarcoma after failure of standard multimodal therapy. Each trial is expected to enroll 37 patients. Finally, Henan Cancer Hospital has registered a single-center, one-armed clinical study to evaluate the efficacy and safety of apatinib as second-line treatment for advanced osteosarcoma and STS. Four out of five of these clinical trials have begun recruiting patients, and the efficacy and safety outcomes may contribute to the eventual approval of apatinib for sarcoma by the U.S. Food and Drug Administration as the second anti-angiogenesis targeted treatment for this disease. The details of these phase I/III trials are summarized in Table 3.

**Table 3:** Ongoing trials of apatinib for sarcoma

Clinical trial identifier	NCT03121846	NCT03064243	NCT03104335	NCT02711007	NCT03163381
Country	China	China	China	China	China
Sponsor	Tianjin Medical University Cancer Institute and Hospital	Shanghai Jiao Tong University Affiliated Sixth People's Hospital	Peking University People's Hospital	Peking University People's Hospital	Henan Cancer Hospital
Phase	II	II	II	II/III	II
Sarcomas type	Stage IV STS patients after failure of traditional chemotherapy	Advanced STS	Advanced STS Patients after failure of traditional therapy	Relapsed and unresectable high-grade osteosarcoma after failure of standard multimodal therapy	Advanced osteosarcoma and STS
Intervention Model	Single arm	Single arm	Single arm	Single arm	Single arm
Masking	None	None	None	None	None
Research center	Multicenter	Single center	Multicenter	Single center	Multicenter
Estimated enrollment	80	53	37	37	40
Primary endpoint	PFS	Six months PFS rate	ORR	PFS, CBR	PFS
Secondary endpoint	DCR, ORR, OS, AEs	-	PFS, OS	OS, ORR, DOR	OS
Start date	May 1, 2017	March 1, 2017	April 1, 2017	March 2016	April 11, 2017
Status	Recruiting	Not yet recruiting	Recruiting	Recruiting	Recruiting

Abbreviations: STS, soft-tissue sarcoma; PFS, progression-free survival; ORR, objective response rate; CBR, clinical benefit rate; DCR, disease control rate; OS, overall survival; AEs, adverse effects; DOR, duration of response.

### Future perspectives

Apatinib is a novel, orally bioavailable small-molecule TK inhibitor of VEGFR-2. Previous studies have shown that apatinib is a promising agent for the treatment of a variety of tumor types, including gastric cancer, NSCLC, triple-negative breast cancer, non-triple-negative breast cancer, and advanced hepatocellular carcinoma, exhibiting improved outcomes and a tolerable safety profile as subsequent-line therapy. Retrospective analyses and case reports have shown that apatinib also exerts a marked effect on sarcomas, with acceptable toxicity, and that it may be superior to other anti-angiogenesis targeted drugs such as pazopanib, sunitinib, and sorafenib. Recently, an interesting study found that the presence of hypertension, proteinuria, or HFS during the first cycle of apatinib treatment correlated with clinical outcomes in gastric cancer patients and was a viable biomarker of antitumor efficacy in metastatic gastric cancer patients [49]. However, no such phenomenon was observed in sarcoma patients, possibly due to fewer cases. Finally, although several phase II/III clinical trials to confirm the efficacy and safety of apatinib for sarcoma are ongoing in China, it may be necessary to establish specific biomarkers to identify appropriate patients for apatinib and to assess the disease prognosis.

## Abbreviations

VEGF: Vascular endothelial growth factor
VEGFRs: Vascular endothelial growth factor receptors
mOS: Median overall survival
TK: Tyrosine kinase
CFDA: China Food and Drug Administration
NSCLC: Non-small cell lung cancer
MTD: Maximum tolerated dose
PR: Partial remission
SD: Stable disease
DCR: Disease control rate
mPFS: Median progression-free survival
ORR: Objective response rate
CBR: Clinical benefit rate
RR: Response rate
mTTP: Median time to progression
AEs: Adverse effects
MFH: Malignant fibrous histiocytoma
ASPS: Alveolar soft part sarcomas
STS: Soft tissue sarcomas
PLS: pleomorphic liposarcomas
STAT3: signal transducer and activator of transcription 3
HFS: hand–foot syndrome
